# Characterization and Functional Analysis of Two Transmembrane C-Type Lectins in Obscure Puffer (*Takifugu obscurus*)

**DOI:** 10.3389/fimmu.2020.00436

**Published:** 2020-03-12

**Authors:** Ying Huang, Yan Shi, Sufei Hu, Ting Wu, Zhe Zhao

**Affiliations:** ^1^College of Oceanography, Hohai University, Nanjing, China; ^2^Postdoctoral Innovation Practice Base, Jiangsu Shuixian Industrial Company Limited, Yangzhou, China; ^3^Guangxi Key Lab for Marine Biotechnology, Guangxi Institute of Oceanography, Guangxi Academy of Sciences, Beihai, China

**Keywords:** *Takifugu obscurus*, C-type lectin, transmembrane region, expression analysis, recombinant protein, antibacterial response

## Abstract

C-type lectins (CTLs) have received widespread attention in animal immune responses. In the present study, two CTLs (*ToCTL1* and *ToCTL2*) were identified from obscure puffer *Takifugu obscurus*. The open reading frames of ToCTL1 and ToCTL2 were 687 and 1,380 bp, respectively. The predicted ToCTL1 and ToCTL2 proteins contained a single transmembrane region and one typical carbohydrate recognition domain (CRD). Quantitative real-time polymerase chain reaction detected *ToCTL1* and *ToCTL2* transcripts in all examined tissues, with high levels in the intestine and kidney, and their expression levels were remarkably altered upon *Vibrio harveyi* and *Aeromonas hydrophila* infection. The recombinant proteins ToCTL1-CRD and ToCTL2-CRD agglutinated the Gram-negative and Gram-positive bacteria in a Ca^2+^-dependent manner. rToCTL1-CRD and rToCTL2-CRD exhibited evident binding activities against seven kinds of bacteria and polysaccharides (lipopolysaccharide and peptidoglycan) in a Ca^2+^-independent manner. Moreover, rToCTL1-CRD and rToCTL2-CRD could inhibit the growth of four types of bacteria *in vitro*. These findings collectively demonstrated that ToCTL1 and ToCTL2 could be involved in host defense against bacterial infection in *T. obscurus*.

## Introduction

Lectins refer to a range of proteins that can selectively recognize and combine carbohydrates, such as lactose, galactose, mannose, N-acetyl glucosamine, and N-acetyl galactosamine, resulting in a non-covalent interaction (1). The interactions of lectin–carbohydrate are recognized as an important component of innate immunity and are used not only for pathogen recognition but also in many different biological processes, such as agglutination, opsonization, phagocytosis, cell adhesion, and complement activation (2, 3). Lectins are widely distributed throughout living organisms, including animals, plants, and microorganisms (4). Animal lectins are generally characterized by at least one carbohydrate recognition domain (CRD), which particularly binds to different sugar moieties present on the surfaces of pathogens (5). On the basis of their CRD structures, motif types, binding specificities, and calcium dependency, animal lectins are broadly classified into 11 primary families, such as C-type, F-type, I-type, L-type, M-type, P-type, R-type, F-box lectins, chitinase-like lectins, ficolins, calnexin, galectins, and intelectins (6).

C-type lectins (CTLs) are the most diverse family of animal lectins that are originally defined as Ca^2+^-dependent sugar-recognition proteins (7). More than 100 years have passed since the discovery of the first CTL, and studies on the properties, structures, and functions of CTLs have been in depth and extensive. Most CTLs contain one or several CRDs consisting of 115–130 amino acids that form a characteristic double-loop structure, disulfide-bond positions, and Ca^2+^-binding sites (8). Each CRD contains 1–4 Ca^2+^ binding sites, among which Ca^2+^ binding site 2 is structurally conserved and is associated with the sugar-binding specificity of CTLs (9). In site 2, a conserved motif Trp-Asn-Asp (WND) and another highly conserved Gln-Pro-Asp (QPD) or Glu-Pro-Asn (EPN) sequence motif are identified. The latter has been proved to bind galactose or mannose (9, 10).

CTLs mediate diverse crucial biological processes, including non-self-recognition, leukocyte adhesion, and rapid stimulation of defense mechanisms to resist harmful microbes (6, 11). To date, CTLs have been identified in a number of fish species and reported to play important roles in immune defense. A CTL is remarkably associated with *Benedenia seriolae* infection levels and is a strong candidate gene for *Benedenia* disease resistance in the Japanese yellowtail/amberjack *Seriola quinqueradiata* (12). In Qihe crucian carp *Carassius auratus*, a novel CTL, Nattectin-like protein (CaNTC), has exhibited strong binding ability with lipopolysaccharide (LPS), peptidoglycan (PGN), and various bacteria. Moreover, CaNTC agglutinates rabbit erythrocytes and three kinds of bacteria in a Ca^2+^-dependent manner (13). A CTL (OnCL11X1) from Nile tilapia (*Oreochromis niloticus*) is upregulated following challenges with *Streptococcus agalactiae* and *Aeromonas hydrophila*. Recombinant OnCL11X1 protein agglutinates the two aforementioned bacteria *in vitro* and promotes phagocytosis by macrophages (14). A CTL family with four member SsCTL4 from the teleost fish black rockfish (*Sebastes schlegelii*) serves as a pattern recognition receptor that not only possesses the inhibitory effect on *Edwardsiella tarda* infection but also serves as targets for virus (infectious spleen and kidney necrosis virus) manipulation of the host defense system (15). However, little is known about the roles of CTLs in the innate immunity of pufferfish. The pattern recognition role of CTLs and the triggered immune responses need to be further studied.

*Takifugu obscurus*, a fish species of economic importance, is becoming increasingly popular in Chinese market due to its desirable flesh quality. However, in the process of breeding, outbreaks of diseases are serious problems that could cause huge economic losses. Therefore, the study on the immune defense mechanism of puffers is of great importance for disease control and prevention. In the present study, two CTL genes in obscure puffer *T. obscurus* (*ToCTL1* and *ToCTL2*) were systematically characterized for the first time. Expression profiles of *ToCTL1* and *ToCTL2* were determined in various tissues of healthy obscure puffer and upon bacterial infection. In addition, recombinant CRDs of ToCTL1 and ToCTL2 were produced using a prokaryotic expression system for analyzing their antibacterial functions *in vitro*. The results of our study will provide the basis for further functional research of CTLs in obscure puffer.

## Materials and Methods

### Experimental Microbes and Animals

*Staphylococcus aureus* [Luria–Bertani (LB) medium, 37°C], *Micrococcus luteus* (LB medium, 37°C), *Bacillus subtilis* (LB medium, 37°C), *B. thuringiensis* (LB medium, 37°C), *Vibrio parahaemolyticus* (LB medium, 28°C), *V. harveyi* [Tryptic Soy Broth (TSB) medium, 28°C], and *A. hydrophila* (TSB medium, 28°C) were stored in our laboratory. *Escherichia coli* LPS and *M. luteus* PGN were purchased from Sigma (St. Louis, MO, USA).

Two hundred *T. obscurus* (body length, 11 ± 2 cm; body mass 25 ± 4 g) individuals were procured from Yangzhong Fugu Aquatic Product (Zhenjiang, Jiangsu Province, China) and transferred to the laboratory (within 3 h) without any physical stress. Before sample collection, the fish were acclimated under laboratory conditions (at 23 ± 1°C) for 1 week in 100 L glass tanks with air-pumped freshwater.

### Sequence Search and Analysis

The CTLs in obscure puffer were identified by searching our previous transcriptome data and National Center for Biotechnology Information (NCBI). TBLASTN similarity searches were performed on the RNA-seq database to identify all CTL gene-related sequences using all available CTL sequences retrieved from the NCBI. The retrieved sequences with all uncharacterized obscure puffer CTL sequences were further analyzed using Open Reading Frame (ORF) Finder (https://www.ncbi.nlm.nih.gov/orffinder/) for the generation of coding sequences. The predicted amino acid sequences were further verified by BLASTP (http://blast.ncbi.nlm.nih.gov/Blast.cgi). On the basis of our obtained sequences, two CTL sequences were synthesized following the instructions of the SMARTer™ RACE cDNA Amplification Kit (Clontech, USA). Gene specific primers (ToCTL1-F:5′-TGGCTGCCCTTTGGCGGGTCCTGTTACT-3′, ToCTL1-R: 5′-GACAGGAAGTAACAGGACCCGCCAAAGG-3, ToCTL2-F: 5′-GCGGACAATGATCGGCTCAGCACAGA-3′, ToCTL2-R:5′-AACTCCACGCAGTCCTGGTTCCTCCC-3′) were used in the synthesis. An Advantage® 2 polymerase chain reaction (PCR) Kit (Clontech, USA) was used for gene cloning. The 25 μL PCR contained 2.5 μL of 10× Advantage 2 PCR buffer, 0.5 μL of 50× dNTP mix, 2.5 μL of 10× Universal Primer A mix, 0.5 μL of 50× Advantage 2 polymerase mix, 1 μL of 3′ or 5′-RACE-ready cDNA, 1 μL of gene-specific primer, and 17 μL of PCR-grade water. PCR was conducted as follows: 5 cycles at 94°C for 30 s and 72°C for 3 min, followed by 5 cycles at 94°C for 30 s, 70°C for 30 s, and 72°C for 3 min, and 20 cycles at 94°C for 30 s, 68°C for 30 s, and 72°C for 3 min. The PCR products were cloned into the pEASY-T3 cloning vector (TransGen Biotech, Beijing, China), from which positive clones were selected for sequencing. The conserved domains and signal peptides of ToCTL1 and ToCTL2 were identified using SMART (http://smart.embl-heidelberg.de/). The theoretical isoelectric point (pI) and molecular weight (MW) were calculated using online ExPASy Compute pI/Mw tool (http://web.expasy.org/compute_pi/). Multiple alignments of protein sequences were performed using ClustalW 2.0 program (http://www.ebi.ac.uk/tools/clustalw2) and GENEDOC software. Phylogenetic and molecular evolutionary analysis was constructed with Molecular Evolutionary Genetics Analysis software (MEGA 7), and 1,000 bootstraps were selected to evaluate reliability (16).

### Immunity Challenge and Tissue Collection

To characterize the expression pattern of *ToCTL1* and *ToCTL2* genes in different tissues of obscure puffer, we collected seven tissue samples (gill, liver, intestine, spleen, muscle, heart, and kidney) from healthy obscure puffer. To evaluate the variation of *ToCTL1* and *ToCTL2* following bacterial stimulation, we conducted the experiment via intraperitoneal injection. A total of 90 fish were injected; two treatment groups and one control group were each injected with 30 fish. The experimental fish were injected with 100 μL of *V. harveyi* (1 × 10^7^ CFU/mL) or *A. hydrophila* (1 × 10^7^ CFU/mL). The control group was injected with sterile 100 μL physiological saline per fish. Intestine, spleen, kidney, and liver tissues were collected in the treated and control groups at 12, 24, 36, and 48 h post-injection from each fish within three replicate tanks. Samples were immediately frozen in liquid nitrogen followed by storage in −80°C until extraction of RNA.

### Total RNA Isolation and cDNA Synthesis

Total RNAs were extracted from the abovementioned tissues with TRIzol® Reagent (Molecular Research Center, Inc., Cincinnati, OH, USA) following the manufacturer's instructions, and genomic DNA was removed by RNase-free DNase I (Takara, Japan). The quality of RNA was evaluated on 1.2% agarose gel by electrophoresis, and the concentration of RNA was measured using a NanoDrop 2000 UV spectrophotometer (Thermo Fisher). The A260/280 ratio of all extracted RNA samples was 1.8–2.1, and the concentration was >200 μg/mL. The first-strand cDNA was synthesized from 1 μg of total RNA using the PrimeScript® 1st Strand cDNA Synthesis Kit (Takara, Japan) with Oligo-d(T) Primer.

### Quantitative Real-Time PCR (qRT-PCR)

qRT-PCR was performed using the SYBR® Premix Ex Taq™ II Kit (Perfect Real Time) (Takara, Japan) on the LightCycler® 96 (Roche, USA). The total PCR reaction volume was 10 μL containing 5 μL of 2 × SYBR® Premix Ex Taq™ II, 1 μL of 1:10 diluted cDNA, 0.4 μL each of the forward and reverse primers (10 μM), and 3.2 μL of ultrapure water. Amplification consisted of an initial denaturation step at 95°C for 60 s and then 40 cycles of 95°C for 5 s, 60°C for 30 s, followed by a melting curve analysis from 60 to 95°C. Gene-specific primers (ToCTL1-qPCR-F: 5′-GCAAGAAGACGGAAGTGAAGA-3′ and ToCTL1-qPCR-R: 5′-GGTTTGGTGGAGAGGAAGATG-3′; ToCTL2-qPCR-F:5′-CCAAGAGAGGAACAACCTACAG-3′ and ToCTL2-qPCR-R:5′-GTGTTCCACGTCCTTGACTAA-3′) were designed on the basis of the CTL mRNA sequences of obscure puffer. β-Actin gene (ToActin-qPCR-F:5′-GCATTGTCACCAACTGGGATG-3′ and ToActin-qPCR-R: 5′-GCAGGACTGGATGCTCCTCT-3′) was used as internal control. All primers were synthesized by Sangon Biotech (Shanghai) Co., Ltd. Three biological replicate RNA samples from healthy and infected tissues were analyzed for gene expression. The relative transcript level of *ToCTL1* and *ToCTL2* was determined using the 2^−ΔΔCt^ threshold cycle (C_T_) method (17). All data are provided in accordance to relative mRNA expressed as mean ± S.D. Data were subjected to one-way ANOVA, with *P* < 0.05 considered statistically significant.

### Recombinant Expression and Purification of ToCTL Proteins

The CRD domains of *ToCTL1* and *ToCTL2* genes were amplified with the primers (ToCTL1-CRD-6P-2-ex-F: 5′-GGATCCCCAGGAATTCCCTGTGACGAGGACTGGCTGCCC-3′ and ToCTL1-CRD-6P-2-ex-R: 5′-GATGCGGCCGCTCGAGTTACTGGCAGATGAAGTAGGTGTA-3′; ToCTL2-CRD-6P-2-ex-F: 5′-GGATCCCCAGGAATTCCCTGCCCCACCGGCTGGAAGAAG-3′ and ToCTL2-CRD-6P-2-ex-R:5′-GATGCGGCCGCTCGAGTTACTCACACATCCAGTTATTTTCG-3′), followed by digestion of the product with *EcoR* I and *Xho* I, subcloned into the expression vector pGEX-6p-2, cut with the same restriction enzymes, and transformed into *E. coli* BL21 (DE3) cells (TransGen Biotech, China). Positive clones were screened by PCR and confirmed by nucleotide sequencing. Subsequently, positive transformants were incubated in 300 mL LB medium (containing 100 μg/mL ampicillin) at 37°C with shaking at 200 rpm for 3 h until OD600 of 0.6–0.8. Isopropyl-β-D-thiogalactoside (IPTG) was then added to the medium to a final concentration of 0.5 mM to induce expression. After culturing at 28°C, 200 rpm for 6 h, the bacterial cells were collected by centrifugation at 6,000 rpm for 10 min and then resuspended in 20 mL of 1× PBS (137 mM NaCl, 2.7 mM KCl, 10 mM Na_2_HPO_4_, 2 mM KH_2_PO_4_, pH7.4) containing 0.1% TritonX-100. Afterward, the collected bacteria were sonicated at 4°C for 40 min in a program of 3 s sonication and 3 s interval (BILON-250Y), and the lysates were centrifuged at 10,000 rpm for 15 min at 4°C to collect the supernatant, which was then purified by glutathione Sepharose 4B chromatography (Gen-Script, USA) following the manufacturer's instruction. Purified proteins were detected by 12% SDS polyacrylamide gel electrophoresis (SDS-PAGE) and observed by Coomassie brilliant blue R250. The concentrations of the rToCTL1-CRD and rToCTL2-CRD proteins were quantified by the BCA (bicinchoninic acid) Protein Assay Kit (Tiangen Biotech, Beijing, China).

### Microorganism Agglutination Activity Assay

Four microorganisms were used to measure the agglutination activity of rToCTL1-CRD and rToCTL2-CRD using an agglutination assay performed as reported previously (18). After cultivation, *S. aureus, V. parahemolyticus, V. harveyi*, and *A. hydrophila* were harvested by centrifugation at 6,000 rpm for 5 min. Microbe pellets were washed three times with tris-buffered saline (TBS, 20 mM Tris-HCl, 150 mM NaCl, pH 7.4) and resuspended to 1 × 10^8^ CFU/mL. In the presence or absence of 10 mM CaCl_2_, a volume of 25 μL bacterial suspension was mixed with 25 μL rToCTL1-CRD or rToCTL2-CRD (100 μg/mL). rGST (100 μg/mL) was mixed with bacterial cells and was used as the negative control. After incubation for 1 h at room temperature, the phenomenon of the agglutination reaction was evaluated by microscopy (Nikon, Japan).

### Microorganism Binding Assay

Four Gram-positive bacteria (*S. aureus, M. luteus, B. subtilis*, and *B. thuringiensis*) and three Gram-negative bacteria (*V. parahemolyticus, V. harveyi*, and *A. hydrophila*) were used to measure the binding activity of rToCTL1-CRD and rToCTL2-CRD using a binding assay described previously with some modifications (19). After cultivation, bacteria were collected by centrifugation at 6,000 rpm for 10 min and then washed three times with TBS. Microorganisms (1 × 10^8^ CFU) were incubated in 1 mL of 100 μg/mL targeted protein with gentle rotation for 1 h at 37°C. Afterward, the pellets of microorganisms were washed three times with TBS by vigorous shaking and eluted with 5% SDS solution with mild shaking. The elution and the final pellets of microorganisms were analyzed by 12% SDS-PAGE. An anti-GST antibody (TransGen, China) was used. As controls, bacterial cells were incubated with rGST and subjected to the same treatments.

### Assay for the Binding of Polysaccharides

Two types of polysaccharides (LPS and PGN) were detected by enzyme-linked immunosorbent assay (ELISA) to detect the direct binding ability of rToCTL1-CRD and rToCTL2-CRD. LPS and PGN were dissolved in water to achieve the concentration of 80 μg /mL and were sonicated for 3 × 15 s on ice. Fifty microliters of polysaccharides (4 μg) were coated to each well of 96-well plates, incubated at 37°C overnight, and heated at 60°C for 30 min. Each well of the plate was blocked with 200 μL of 5% bovine serum albumin (BSA) in TBS at 37°C for 2 h and then washed thrice with TBS. One hundred microliters of various concentrations of recombinant protein (0.78, 1.56, 3.125, 6.25, 12.5, and 25 μg/mL dissolved in TBS) were added to the wells. The plate was incubated at 37°C for 3 h and then washed three times. Rabbit monoclonal anti-GST antibody (1:2,000 dilution in TBS with 0.1 mg/mL BSA) was added to each well and incubated at 37°C for 2 h. The plate was washed thrice as described previously and incubated with peroxidase-conjugated goat anti-rabbit IgG secondary antibodies (1:5,000 dilution in TBS with 0.1 mg/mL BSA) at 37°C for 1 h. The plate was washed thrice again and developed with 100 μL of 0.01% 3,3′,5,5′-tetramethylbenzidine (Sigma–Aldrich, USA). The reaction was stopped with 2 M H_2_SO_4_, and the absorbance was recorded at 450 nm. All assays were repeated thrice. For the control, rGST was used instead of rToCTL1-CRD and rToCTL2-CRD.

### Antimicrobial Activity Assay

The effect of rToCTL1-CRD and rToCTL2-CRD on the growth curves of a Gram-positive bacterium (*S. aureus*) and three Gram-negative bacteria (*V. parahemolyticus, V. harveyi*, and *A. hydrophila*) was investigated (20). Ten microliters of bacteria grown overnight were transferred into 5 mL of LB medium. The final concentration of purified rToCTL1-CRD or rToCTL2-CRD was 100 μg/mL. An equal volume of GST-tag protein was used as the negative control. Each sample was incubated at 37°C with shaking at 200 rpm, and OD600 was determined every 1 h. All assays were repeated thrice.

## Result

### Characterization of ToCTL1 and ToCTL2 cDNA

**Figure 1:**
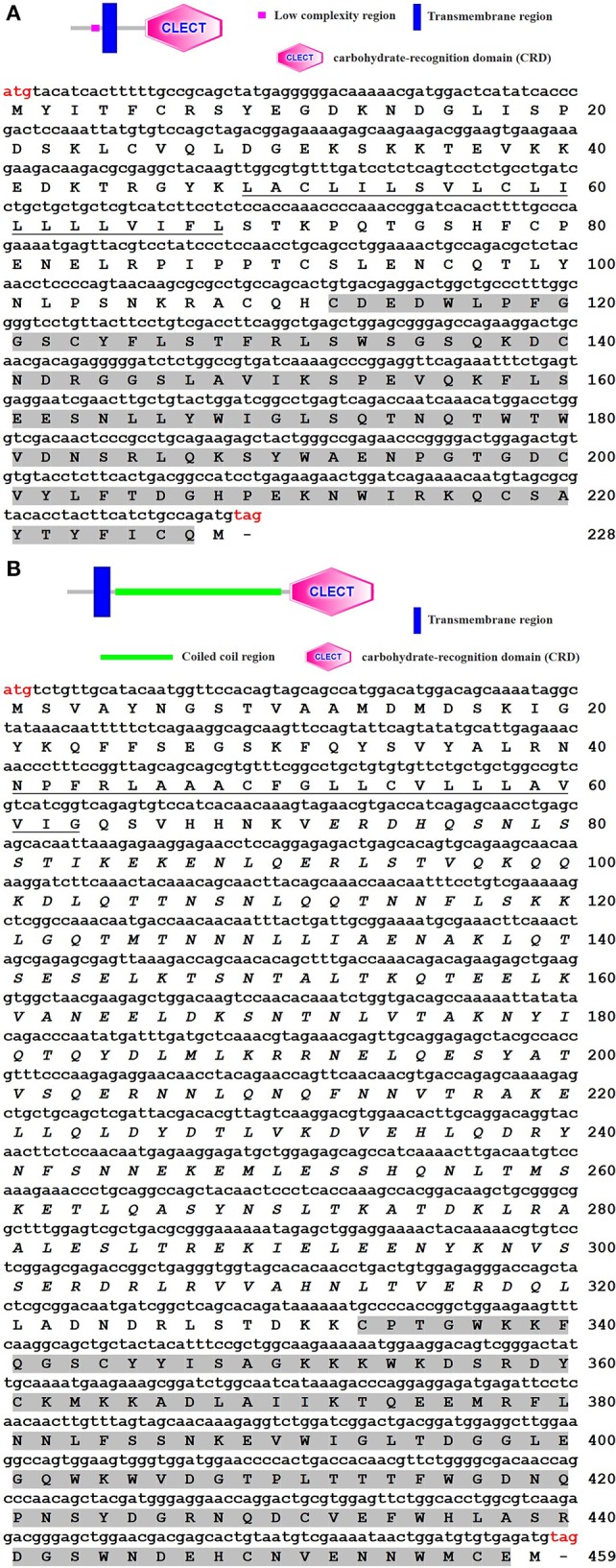
Structures (above) and cloned cDNA sequences (below) of ToCTL1 **(A)** and ToCTL2 **(B)**. The start codon (ATG) and stop codon (TAG) are marked in red. The transmembrane regions are underlined, and the CRDs are shaded in gray.

The cDNA sequences of *ToCTL1* and *ToCTL2* were obtained via transcriptome sequencing of obscure puffer liver and confirmed by PCR and sequencing. The ORF of *ToCTL1* was 687 bp encoding a 228-amino acid protein (Figure 1A). A 20-amino acid transmembrane region and one CRD (residues 112–227) were predicted in the deduced protein. The mature ToCTL1 peptide had a theoretical MW of 26.08 kDa and a pI of 6.79. *ToCTL2* contained an ORF of 1,380 bp encoding a putative protein of 459 amino acid residues with predicted MW and pI of 52.92 kDa and 6.31, respectively. ToCTL2 contained a transmembrane region (residues 41–63) and a CRD domain (residues 333–458) (Figure 1B).

### Sequence Analysis of ToCTL1 and ToCTL2

**Figure 2:**
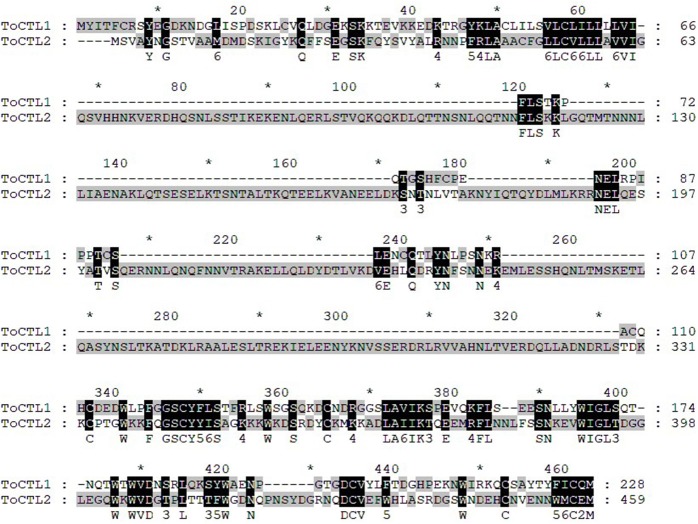
Multiple alignments of *T. obscurus* ToCTL1 and ToCTL2 protein sequences. Consensus sequences of ToCTL1 and ToCTL2 are highlighted with shaded background and are shown below the alignment.

**Figure 3:**
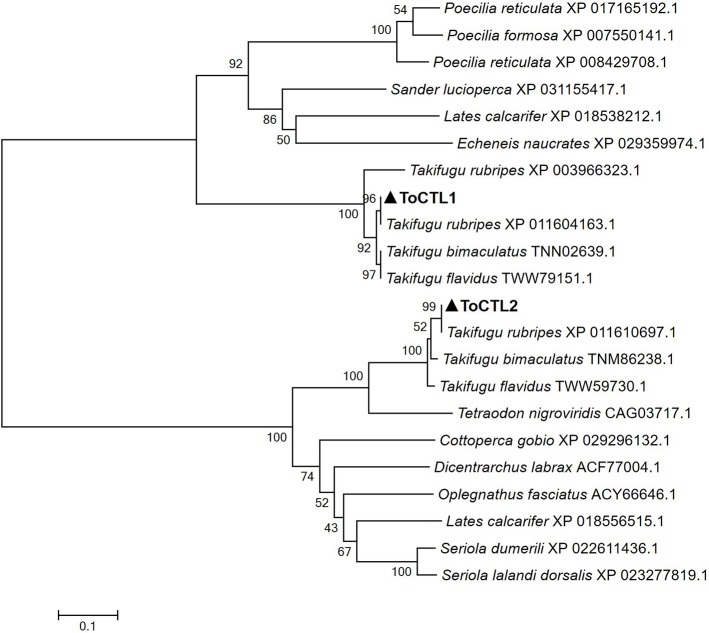
Phylogenetic analysis of CTL proteins from pufferfish and other fish. ToCTL1 with other CTL proteins included the following: CTL homolog from *T. rubripes, T. bimaculatus, Sander lucioperca*; Asialoglycoprotein receptor 2 from *T. flavidus*; early activation antigen CD69-like isoform X from *T. rubripes, Poecilia reticulata, Lates calcarifer, Echeneis naucrates, P. reticulata, Poecilia formosa*. ToCTL2 with other CTL proteins included the following: CTL homolog from *T. rubripes, T. bimaculatus, T. flavidus, T. nigroviridis, Dicentrarchus labrax, Oplegnathus fasciatus*; CD209 antigen-like from *Seriola dumerili, L. calcarifer, Seriola lalandi dorsalis, Cottoperca gobio*. ToCTL1 and ToCTL2 are labeled with solid triangle.

Multiple alignment analysis showed that ToCTL1 and ToCTL2 were different in amino acid composition although ToCTL1 and ToCTL2 belonged to the same species (Figure 2). BLASTP analysis using ToCTL1 as template against the available databases identified close matches in the CTL homologs of *T. rubripes* (XP_011604163.1), *T. bimaculatus* (TNN02639.1), and *T. flavidus* (TWW79151.1), which shared 100, 98.3, and 91.4% overall amino acid sequence identities, respectively, with ToCTL1. ToCTL2 was similar to CTL protein from *T. rubripes* (XP_011610697.1), with 100% identity, 97.4% identity with CTL from *T. bimaculatus* (TNM86238.1), 97% identity with CTL from *T. flavidus* (TWW59730.1), and 74.3% identity with CTL homolog from *Tetraodon nigroviridis* (CAG03717.1). Phylogenetic analysis showed that CTL homolog proteins could be divided into two distinct clades. Sequences of ToCTL1 and ToCTL2 were remarkably different and belonged to different groups (Figure 3).

### Tissue Distribution of ToCTL1 and ToCTL2 in Healthy Obscure Puffer

**Figure 4:**
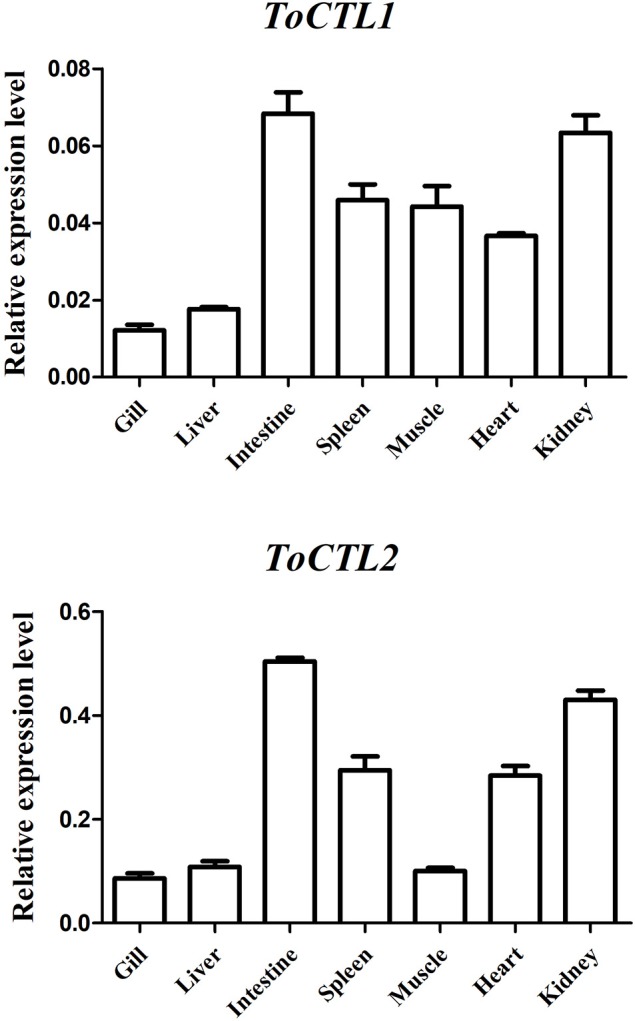
Gene expression analysis of *ToCTL1* and *ToCTL2* in different healthy tissues of obscure puffer. *ToCTL1* and *ToCTL2* transcript levels in the gill, liver, intestine, spleen, muscle, heart, and kidney of *T. obscurus* were determined by qRT-PCR. β-actin was used as the reference gene. The error bars represent the standard deviation of mean ± S.D. (*N* = 5).

The transcripts of *ToCTL1* and *ToCTL2* were tested by qRT-PCR in several tissues from uninfected obscure puffer (Figure 4). The expression level of *ToCTL2* in healthy pufferfish was considerably higher than that of *ToCTL1*. *ToCTL1* expressed in the intestine, kidney, spleen, muscle, and heart was at high levels, whereas *ToCTL1* expressed in the liver and gill was at low levels. Apart from the muscle, the transcript profile of *ToCTL2* was similar to that of *ToCTL1*, indicating that *ToCTL1* and *ToCTL2* serve as important molecules expressed in a range of tissues.

### Time-Course Expression Profiles of ToCTL1 and ToCTL2 After Immune Challenge

**Figure 5:**
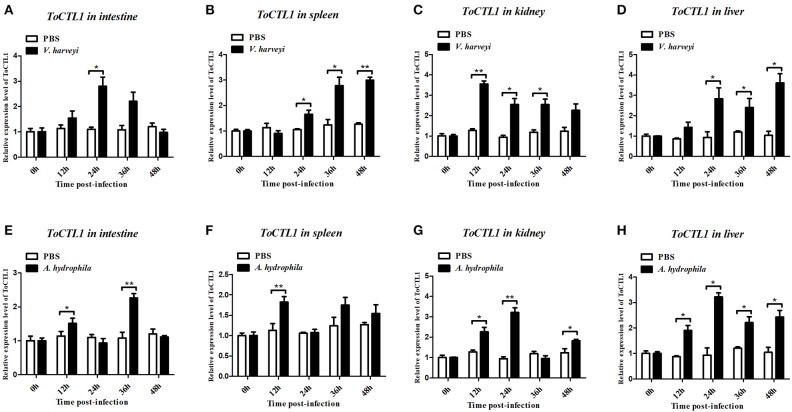
Temporal mRNA expression of *ToCTL1* was determined by qRT-PCR in the intestine **(A,E)**, spleen **(B,F)**, kidney **(C,G)**, and liver **(D,H)** of *T. obscurus* after *V. harveyi* and *A. hydrophila* infection. The mRNA level of *ToCTL1* gene was normalized against the housekeeping gene β-actin and expressed relative to the PBS control group, which was used as 1-fold control. Vertical bars represent mean ± S.D. (*N* = 5). The statistical *p-*values were calculated by one-way ANOVA. Differences were considered significant at *p < 0.05 and highly significant at **p < 0.01.

**Figure 6:**
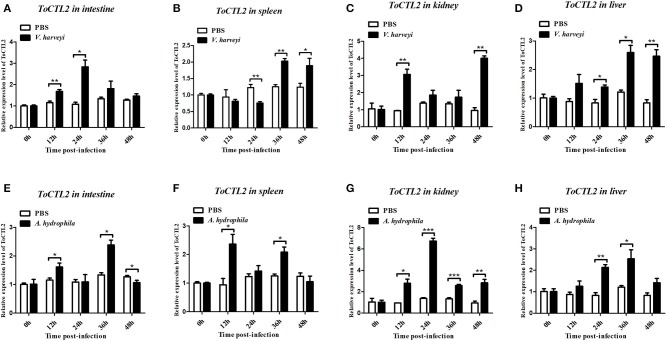
Temporal mRNA expression of *ToCTL2* was determined by qRT-PCR in the intestine **(A,E)**, spleen **(B,F)**, kidney **(C,G)**, and liver **(D,H)** of *T. obscurus* after *V. harveyi* and *A. hydrophila* infection. The mRNA level of *ToCTL2* gene was normalized against the housekeeping gene β-actin and expressed relative to the PBS control group, which was used as 1-fold control. Vertical bars represent mean ± S.D. (*N* = 5). The statistical *p-*values were calculated by one-way ANOVA. Differences were considered significant at *p < 0.05 and highly significant at **p < 0.01, ***p < 0.001.

To characterize the innate immune responses of *ToCTL1* and *ToCTL2* to bacterial infection in the host, we conducted *V. harveyi* and *A. hydrophila* challenge. *ToCTL1* and *ToCTL2* cDNAs were remarkably upregulated in the four tissues upon immune challenge but showed no remarkable change in their expression in the PBS-treated group. After *V. harveyi* challenge, the expression levels of *ToCTL1* increased to its highest level at 24, 12, or 48 h with 2.8-, 3.5-, or 3.6-fold compared with those in the controls in the intestine (Figure 5A), kidney (Figure 5C), or liver (Figure 5D), respectively. In the spleen, *ToCTL1* mRNA was continuously upregulated from 24 to 48 h (Figure 5B). After challenge with *A. hydrophila, ToCTL1* mRNA in the intestine and spleen began to increase within 12 h, decreased at 24 h, and then reached maximum at 36 h (Figures 5E,F), whereas that in the kidney and liver, *ToCTL1* expression levels quickly increased to the highest level at 24 h and then slowly decreased at 36 h (Figures 5G,H). After *V. harveyi* challenge, the highest expression of *ToCTL2* in the intestine (Figure 6A) and kidney (Figure 6C) was observed at 24 and 48 h post injection, respectively. The *ToCTL2* expression reached the highest level at 36 h in the spleen (Figure 6B) and liver (Figure 6D). In the *A. hydrophila*-stimulated group, *ToCTL2* in the intestine (Figure 6E) and spleen (Figure 6F) increased at 12 and 36 h, whereas that in the kidney (Figure 6G) and liver (Figure 6H), *ToCTL2* was upregulated from 12 to 24 h and reached its peak at 24 and 36 h, respectively. These results suggested that obscure puffer *ToCTL1* and *ToCTL2* serve as immune factors involved in pathogen *V. harveyi* and *A. hydrophila* defense.

### Recombinant Expression and Purification of ToCTL1-CRD and ToCTL2-CRD

**Figure 7:**
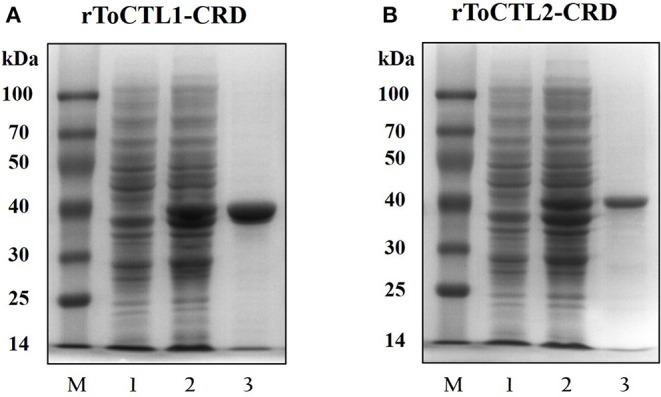
Recombinant expression and purification analysis of rToCTL1-CRD **(A)** and rToCTL1-CRD **(B)**. Lane M, markers; Lane 1, the bacteria liquid before IPTG induction; Lane 2, the pGEX-6p-2-rToCTL1-CRD or pGEX-6p-2-rToCTL2-CRD was induced with 0.5 mM IPTG at 28°C for 6 h; Lane 3, purified rToCTL1-CRD or rToCTL2-CRD fusion protein.

Recombinant ToCTL1-CRD and ToCTL2-CRD were expressed in *E. coli* BL21 (DE3) and purified with glutathione resin. ToCTL1-CRD and ToCTL2-CRD comprised a mature peptide (theoretical MW: 13.43 kDa for ToCTL1-CRD and 14.75 kDa for ToCTL2-CRD) and ~26 kDa of GST-tag. The predicted MW of ToCTL1-CRD or ToCTL2-CRD (~39.43 or 40.75 kDa) agreed with the size of a major band that appeared in either of the purified protein lanes (Figure 7).

### Microbial Agglutination and Binding of rToCTL1-CRD and rToCTL2-CRD

**Figure 8:**
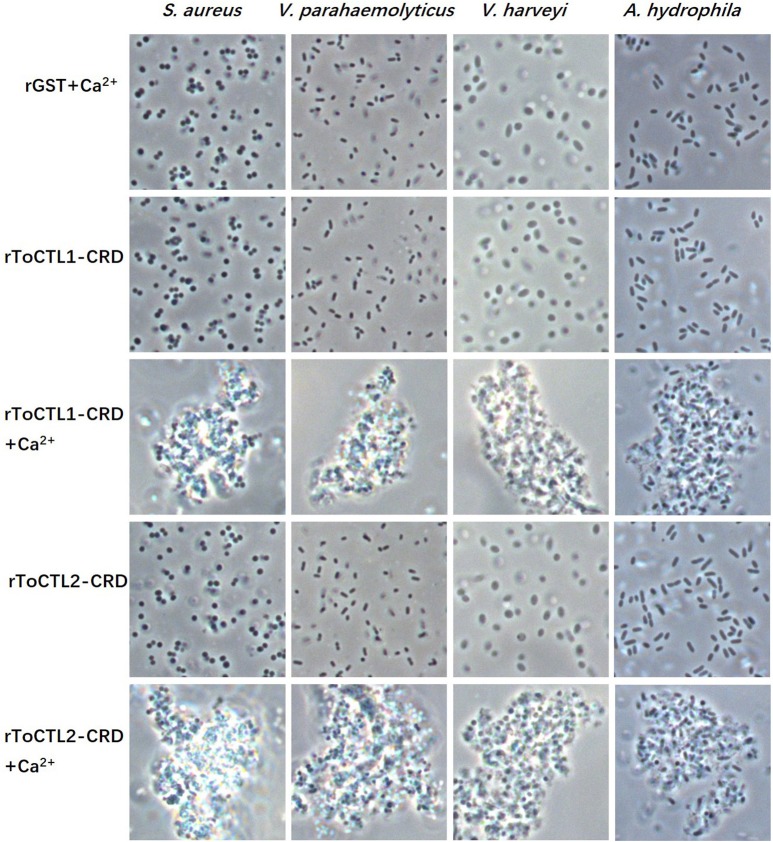
Agglutination activity of recombinant ToCTL1-CRD and ToCTL2-CRD. *S. aureus, V. parahaemolyticus, V. harveyi*, and *A. hydrophila* were incubated with rToCTL1-CRD or rToCTL2-CRD in the absence or presence of Ca^2+^. rGST was mixed with bacterial cells and was used as the negative control. The agglutination reaction was observed with a microscope.

rToCTL1-CRD and rToCTL2-CRD possess antimicrobial activities; hence, their agglutination activities were investigated. Results demonstrated that rToCTL1-CRD and rToCTL2-CRD caused strong agglutination of one Gram-positive bacteria (*S. aureus*) and three Gram-negative bacteria (*V. parahaemolyticus, V. harveyi*, and *A. hydrophila*) in the presence of Ca^2+^. Under the same condition, rGST did not exhibit any agglutinating ability to the test bacteria (Figure 8).

**Figure 9:**
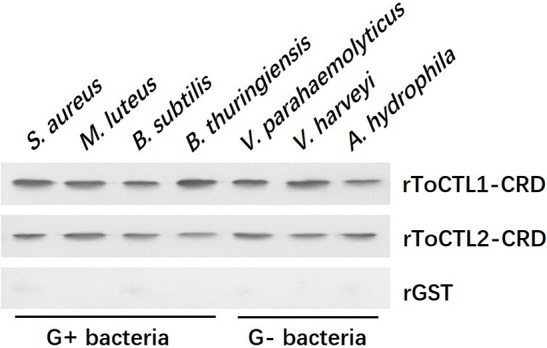
Binding activity of rToCTL1-CRD and rToCTL2-CRD to microorganisms. Seven kinds of bacteria (G+ bacteria *S. aureus, M. luteus, B. subtilis, B. thuringiensis*, and G– bacteria *V. parahaemolyticus, V. harveyi, A. hydrophila*) were incubated with rToCTL1-CRD or rToCTL2-CRD and then washed three times with TBS and subjected to elution with 5% SDS. The binding activity was confirmed by Western blotting analysis. rToCTL1-CRD and rToCTL2-CRD were detected by monoclonal antibody against GST. The binding activity of GST protein was tested and used as the negative control.

To clarify the potential antibacterial mechanism of rToCTL1-CRD and rToCTL2-CRD, we conducted a microorganism-binding assay to determine whether these two proteins exhibit binding activities to microbial cells. Results revealed that rToCTL1-CRD and rToCTL2-CRD were firmly attached to the following bacteria: four Gram-positive bacteria (*S. aureus, M. luteus, B. subtilis*, and *B. thuringiensis*) and three Gram-negative bacteria (*V. parahaemolyticus, V. harveyi*, and *A. hydrophila*) (Figure 9). rToCTL1-CRD and rToCTL2-CRD exhibited broad binding activities to these microorganisms.

### PAMP Binding of rToCTL1-CRD and rToCTL2-CRD

**Figure 10:**
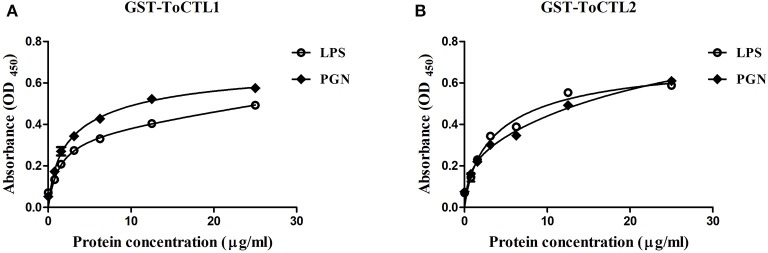
Microbial polysaccharide-binding activity was investigated by ELISA. LPS from *E. coli* or PGN from *M. luteus* was used to coat plates. rToCTL1-CRD **(A)** and rToCTL2-CRD **(B)** were diluted into different concentrations (0.78, 1.56, 3.125, 6.25, 12.5, and 25 μg/mL) and added to the polysaccharide-coated plates. After incubation with anti-GST antiserum, the interaction was detected with anti-rabbit IgG secondary antibody at 450 nm. The control wells were filled with rGST protein. Results were obtained based on three independent repeats.

To characterize the polysaccharide recognition capacities of rToCTL1-CRD and rToCTL2-CRD, we used ELISA to measure the binding activities to LPS and PGN. Results showed that compared with control, rToCTL1-CRD and rToCTL2-CRD exhibited evident binding abilities to LPS, and PGN increased with increasing concentrations of purified proteins (from 0 to 30 μg/mL). Moreover, rToCTL1-CRD and rToCTL2-CRD might show different binding activities to certain surface molecules. The binding activity of rToCTL1-CRD to PGN was relatively stronger than that of LPS (Figure 10A), whereas rToCTL2-CRD showed similar binding activity to LPS and PGN (Figure 10B). By contrast, rGST (as a control) did not exhibit any binding activity to polysaccharides (data not shown).

### Bacterial Growth Inhibition Assay

**Figure 11:**
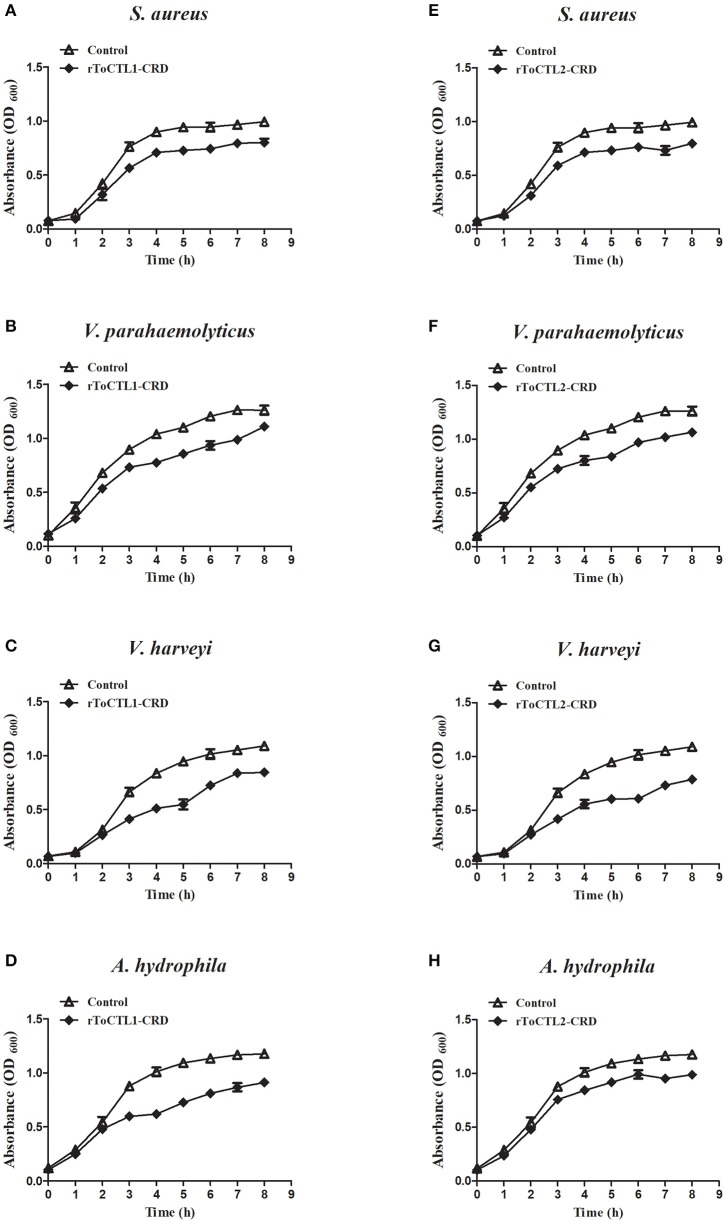
Microorganism growth inhibitory activities of rToCTL1-CRD and rToCTL2-CRD protein. Ten microliters of overnight *S. aureus* **(A,E)**, *V. parahaemolyticus* **(B,F)**, *V. harveyi* **(C,G)**, or *A. hydrophila* **(D,H)** culture was inoculated in LB medium with rToCTL1-CRD (100 μg/mL) or rToCTL2-CRD (100 μg/mL). Bacterial growth was evaluated by measuring the absorbance at 600 nm every 1 h. rGST protein was used as the control. Each experiment was repeated three times.

To detect whether rToCTL1-CRD and rToCTL2-CRD have the ability to retard bacterial growth *in vitro*, we performed antimicrobial activity assays. Results revealed that rToCTL1-CRD and rToCTL2-CRD (100 μg/mL) inhibited the growth of *S. aureus* (Figures 11A,E), *V. parahaemolyticus* (Figures 11B,F), *V. harveyi* (Figures 11C,G), and *A. hydrophila* (Figures 11D,H). The bacteria had slow growth without an evident difference in all experimental groups at 0–2 h, whereas rToCTL1-CRD and rToCTL2-CRD suppressed the microbial growth at 3–8 h (logarithmic phase). rGST, which was used as a control protein, had no effect on bacterial growth. Therefore, rToCTL1-CRD and rToCTL2-CRD had antibacterial functions.

## Discussion

CTLs play pivotal roles in the immune system by interacting with pathogens (9, 10). CTLs have been extensively studied in fish, but they remain poorly investigated in pufferfish. In the present study, two CTL genes (*ToCTL1* and *ToCTL2*) in obscure puffer were characterized for the first time, supported by structural and functional lines of evidence. Sequence comparison showed that the predicted ToCTL1 and ToCTL2 proteins primarily contained two regions: a single transmembrane domain and one typical CRD domain. These structural features were found conserved in the following fish species: *T. macdonaldi* (21), *S. schlegelii* (15), *Lates calcarifer* (22), *Plecoglossus altivelis* (23), and *Salmo salar* (1). However, the CTL receptors can be also soluble proteins (24). CRD is the identified functional region of CTLs, with the participation of Ca^2+^, to selectively identify the sugar structure on the surface of various pathogens (25). The CRD domain of ToCTL1 and ToCTL2 comprised 116 and 126 amino acid residues, respectively, including four conserved cysteine residues associated with the disulfide bond fold. The CRDs with EPN motif preferentially bind mannose, whereas the QPD tripeptide motif binds galactose (9, 10). CTLs from *C. auratus, Cyprinus carpio*, and *Danio rerio* contain EPN motifs, and a CTL from *Xiphophorus maculatus* contains a QPD motif (13). ToCTL1 and ToCTL2 did not have typical QPD and EPN motifs, suggesting that ToCTL1 and ToCTL2 might have different functions in binding carbohydrates. The phylogenetic analysis provided insights into the evolution of CTL genes. ToCTL1 and ToCTL2 were clustered into two clades, but ToCTL1 and ToCTL2 were most closely related to the corresponding pufferfish CTLs, which agreed with features found in the sequence, indicating their strong relationships with pufferfish.

Tissue distribution analysis showed that *ToCTL1* and *ToCTL2* were expressed in all studied tissues of healthy obscure puffer, which was consistent with other reported CTLs (13–15, 21). The mRNA transcription of *CLEC17A* gene from *Totoaba macdonaldi* showed a constitutive expression in all examined tissues, and its expression levels were the highest in the head-kidney, liver, spleen, and skin (21). *OnCL11X1* from *O. niloticus* was highly expressed in the liver and widely exhibited in the kidney, intestines, and spleen (14). In *S. schlegelii, SsCTL4* expression was distributed in the brain, muscle, kidney, spleen, intestine, heart, gills, and liver with an increasing order (15). *CaNTC* from *C. auratus* was the most abundant in liver, followed by the head-kidney and spleen (13). Despite the wide tissue distribution, *ToCTL1* and *ToCTL2* had higher expression levels in one mucosal tissue (intestine) and two classical immune tissues (kidney and spleen) than those in other tissues. The mucosal surfaces of aquatic species (skin, gills, and intestines) are the first line of host defense, constantly interacting with various pathogens in the aquatic environment (26). During infection, the pathogen must cross the mucosal surface barrier to enter the host body smoothly and establish effective colonization and replication (27). The high expression levels of *ToCTL1* and *ToCTL2* in mucosal and immune tissues might be related to the important roles they play in immune responses.

Therefore, we continued to investigate the expression patterns of *ToCTL1* and *ToCTL2* in obscure puffer intestine, spleen, kidney, and liver after *V. harveyi* and *A. hydrophila* infection. *V. harveyi* is a Gram-negative, halophilic bacterium that coexists in the intestinal flora of animals and is a primary and opportunistic pathogen of commercially farmed marine vertebrate species (28). In large yellow croaker *Larimichthys crocea, LcNTC* expression was highly expressed in the liver, spleen, and head-kidney following infection with *V. parahaemolyticus* (29). The expression levels of *SsCTL4* in the head-kidney, liver, and spleen of Black rockfish were remarkably enhanced after being challenged with *Vibrio anguillarum* (15). As a ubiquitous bacterium, *A. hydrophila* is considered one of the most important pathogens for human and lower vertebrates, including fish and amphibians (30, 31). After being challenged with *A. hydrophila*, the temporal expression levels of *CaNTC* in Qihe crucian carp were evidently upregulated in the liver, spleen, and head-kidney (13); the *OnCL11X1* expression in Nile tilapia was upregulated in the spleen and anterior kidney (14). The expression levels of *ToCTL1* and *ToCTL2* were remarkably upregulated in a time-dependent manner by *V. harveyi* and *A. hydrophila* infection in all four tissues examined. Hence, information on *ToCTL1* and *ToCTL2* mRNA distribution in different tissues and their response to immune stimulus are important to understand their particular roles in immunity.

Agglutination activity is a classical characteristic of lectins. With the ability to recognize and non-covalently bind specific sugar moieties, lectins can agglutinate cells by binding to glycoproteins and sugar conjugates on the surface of the cell. However, few studies have been conducted on the function of lectins in fish. In the present study, we successfully obtained the recombinant proteins ToCTL1-CRD and ToCTL2-CRD through the prokaryotic expression system. From the agglutination experiment, rToCTL1-CRD and rToCTL2-CRD were able to agglutinate bacteria in a Ca^2+^-dependent manner. In the case of rSsCTL4, rSsCTL4 caused agglutination of Gram-negative and Gram-positive bacteria, i.e., *E. tarda, V. anguillarum, V. vulnificu*, and *S. aureus* in the presence of Ca^2+^ (15). Recombinant OnCL11X1 protein was able to agglutinate *S. agalactiae* and *A. hydrophila* in a Ca^2+^-dependent manner *in vitro* (14). rCaNTC displayed agglutinating activity against Gram-negative bacteria (*E. coli* and *A. hydrophila*) and Gram-positive bacteria (*S. aureus*) in the presence of Ca^2+^ (13). In ayu *P. altivelis*, rPaCTLRC agglutinated three Gram-positive bacteria (*Listeria monocytogenes, S. aureus*, and *Streptococcus iniae*) and four Gram-negative bacteria (*A. hydrophila, E. coli, V. anguillarum*, and *V. parahaemolyticus*) *in vitro* (23). CTLs are involved in host immune defense, and agglutinating activity against pathogens indicates the relative binding affinity of CTLs to pathogens.

The combination experiment of bacteria and recombinant proteins demonstrated that rToCTL1-CRD and rToCTL2-CRD could combine with four types of Gram-positive bacteria and three types of Gram-negative bacteria in the absence of Ca^2+^. The result showed that rToCTL1-CRD and rToCTL2-CRD might play a pattern recognition role through interaction with bacteria. According to previous reports, Nile tilapia rCL11X1 could combine with Gram-positive bacterium (*S. agalactiae*) and Gram-negative bacterium (*A. hydrophila*) in the presence of Ca^2+^ (14); *S. schlegelii* rSsCTL4 was able to bind to *E. tarda* and *V. anguillarum* in a Ca^2+^-dependent manner (15). The relatively strong affinity of *Carassius auratus* rCaNTC was observed against *A. hydrophila, E. coli*, and *S. aureus* compared with *V. anguillarum, B. subtilis*, and *Micrococcus lysodeikticus* in a Ca^2+^-independent manner (13). Certain CTLs bind to bacteria in the absence of Ca^2+^, whereas other CTLs can bind to bacteria in the presence of Ca^2+^, suggesting that the presence of Ca^2+^ is not necessary for CTLs to bind to bacteria. To date, the relationship between the conserved Ca^2+^-binding motif and Ca^2+^-dependent activity remains unclear and requires further study. In particular, the primary function of CRD domain is to recognize antigens with carbohydrate residues located on the surface of pathogens. In previous studies, rPaCTLRC bound to LPS, PGN, D-mannose, D-galactose, L-fucose, and N-acetyl-D-glucosamine (GlcNAc), exhibiting a relative binding strength to D-mannose and PGN (23). rCaNTC could bind to polysaccharides, including LPS and PGN, exhibiting a dose-dependent manner (13). In the present study, rToCTL1-CRD and rToCTL2-CRD could bind LPS and PGN in a dose-dependent manner. LPS and PGN were the primary components of the outer membranes of Gram-negative and Gram-positive bacteria, respectively. Considering that the recognition of PAMPs on the surface of microbe is the first line of defense, the broad binding spectrum of rToCTL1-CRD and rToCTL2-CRD toward bacteria and polysaccharides play key roles in the immune defense against various pathogenic bacteria. Moreover, rToCTL1-CRD and rToCTL2-CRD could inhibit the growth of four bacteria *in vitro*. We speculate that CRDs agglutinate bacteria by binding to polysaccharides on the surface of bacteria, thereby inhibiting bacterial growth. This finding confirmed the important role of CTLs in eliminating invading non-self.

In conclusion, two novel C-type lectins (*ToCTL1* and *ToCTL2*) were successfully identified and characterized from obscure puffer *T. obscurus*. The mRNA encoding *ToCTL1* and *ToCTL2* was predominantly produced in the intestine and kidney. Following bacterial challenges, the expression levels of *ToCTL1* and *ToCTL2* were remarkably upregulated *in vivo*. Moreover, rToCTL1-CRD and rToCTL2-CRD agglutinated bacteria, bound to carbohydrates and a variety of bacteria, and inhibited bacterial growth *in vitro*. Collectively, these findings indicated that ToCTL1 and ToCTL2 might be implicated in *T. obscurus* innate immunity.

## Data Availability Statement

The datasets generated for this study are available on request to the corresponding author.

## Ethics Statement

The protocol was approved by the Ethics Committee of Experimental Animals at Hohai University.

## Author Contributions

YH and SH carried out the experiments. YH, YS, and TW designed the experiments and analyzed the data. YH contributed reagents and materials. YH and ZZ wrote the manuscript. All authors gave final approval for publication.

### Conflict of Interest

YH and TW were employed by Jiangsu Shuixian Industrial Company Limited. The remaining authors declare that the research was conducted in the absence of any commercial or financial relationships that could be construed as a potential conflict of interest.
